# Right Superior Ophthalmic Vein Thrombosis Induced by Pansinusitis

**DOI:** 10.7759/cureus.34857

**Published:** 2023-02-11

**Authors:** Ahmed Elsaadawy, Binita Panchasara, Abhijeet Yadav

**Affiliations:** 1 Department of Psychiatry, Fairfield General Hospital, Bury, GBR; 2 Department of Ophthalmology, West Suffolk Hospital, Bury St Edmunds, GBR

**Keywords:** cavernous sinus thrombosis, pre-septal cellulitis, orbital cellulitis, pansinusitis, superior ophthalmic vein thrombosis

## Abstract

Superior ophthalmic vein thrombosis (SOVT) is a rare, sight-threatening condition. It can be clinically challenging to distinguish from pre-septal cellulitis or cavernous sinus thrombosis. Imaging is often a key to identifying SOVT, and multi-disciplinary input is paramount to ensuring the optimum outcome. This often involves the paediatricians, ophthalmologists and paediatric neurologists if necessary. We report a case of a young boy with right SOVT that had initially been diagnosed as pre-septal cellulitis. A contrast-enhanced computed tomography scan was performed, as the patient developed limited eye movement on elevation, which showed dilatation of the right ophthalmic vein with pansinusitis. He was successfully treated with anticoagulation and antibiotics.

## Introduction

In the acute setting, orbital signs are often attributed to common pathologies such as peri-orbital cellulitis or sinus infections. This can result in an oversight of rarer conditions such as superior ophthalmic vein thrombosis (SOVT), which can have significant implications for patients. SOVT is caused by orbital congestion, which can occur as a result of infectious or non-infectious causes. The former includes orbital cellulitis, which may present with proptosis, diplopia and visual impairment, and can precipitate a SOVT or cavernous sinus thrombosis [1-3].

A key factor behind the successful management of such cases is early intervention and collaboration between the paediatric neurologists, paediatricians and ophthalmologists. This report aims to highlight presenting features of this condition in a younger age group and emphasise the importance of a multi-disciplinary approach to its management.

## Case presentation

A healthy boy in his first decade of life with a background of previously treated exotropia, amblyopia and attention-deficit/hyperactivity disorder (ADHD) was brought to the emergency department by his mother. He had developed a low-grade fever and upper respiratory tract symptoms over the past week and had suffered a minor injury in which he hit his face onto a door at home. It was thought that the latter was the cause of his eyelid swelling; therefore, he was initially discharged with safety netting advice. The child returned the next day with worsening swelling, right-sided peri-orbital pain and tenderness along the medial canthus. At the time, there was no visual impairment or any neurological impairment detected on examination. His body temperature was 37.6◦C, blood pressure was 104/59 mmHg, heart rate was 75 bpm and capillary refill time was under 2 seconds.

Basic eye assessment in the emergency department revealed a Snellen visual acuity of 6/7.5 on the right eye (amblyopic eye) and 6/5 on the left eye with white conjunctivae, reactive pupils and normal eye movements. He was later seen by an ophthalmologist who agreed that this be treated as a pre-septal cellulitis, as his eye movements were full with no clinical signs to suggest deeper involvement.

Despite admission and treatment with intravenous antibiotics, the lid swelling continued to worsen without conjunctival injection, and he gradually developed limitation and pain on attempted elevation of the right eye.

Investigations

**Figure 1:**
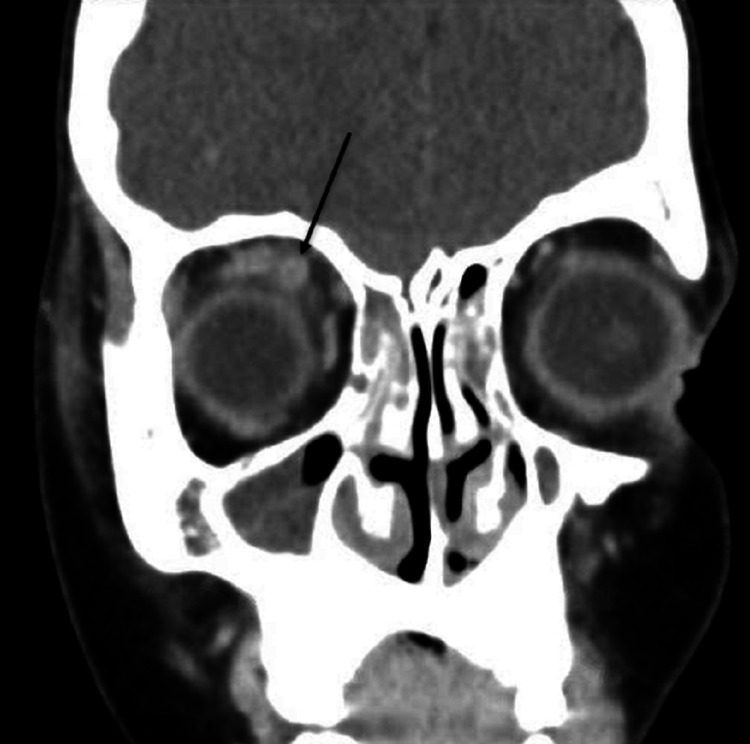
Post-contrast CT (coronal view) showing right superior ophthalmic vein thrombosis with vein enlargement in comparison to the left side with sinusitis.

**Figure 2:**
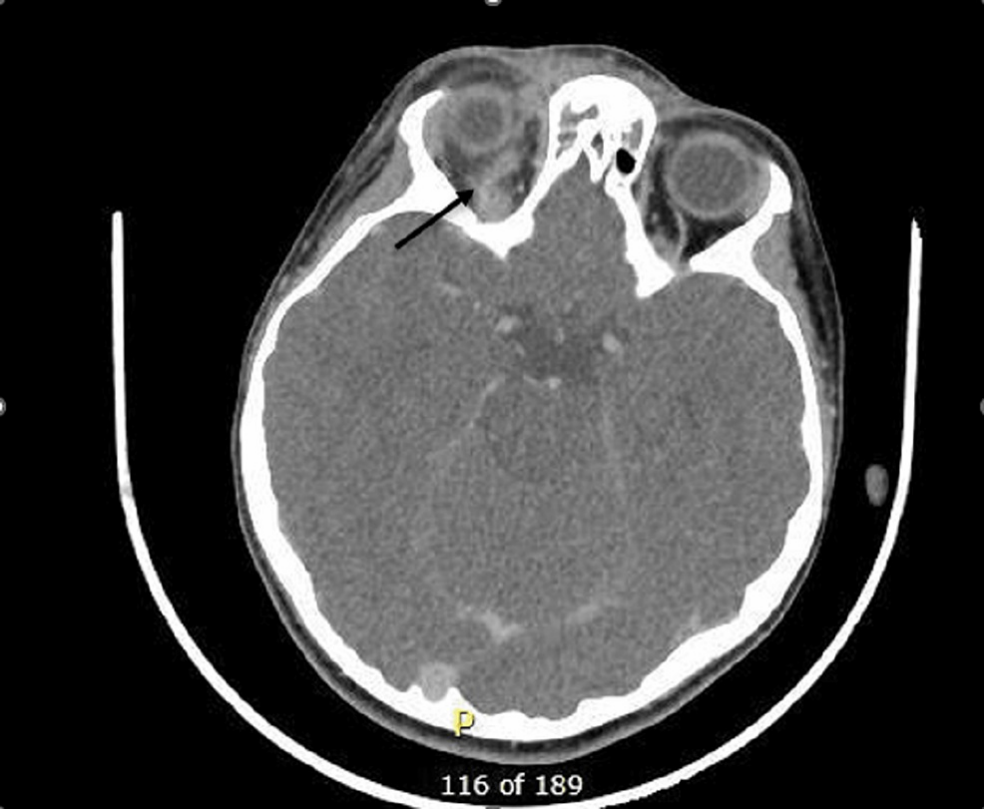
Post-contrast CT (axial view) showing right superior ophthalmic vein thrombosis with vein enlargement in comparison to the left side.

In this case, before the decision was taken to perform imaging, the patient was being treated for pre-septal cellulitis with intravenous antibiotics; however, his symptoms continued to deteriorate. A contrast-enhanced computed tomography scan of the orbits, sinuses and brain was performed, which revealed pansinusitis with a collection adjacent to the right frontal sinus in addition to enlargement of the right superior ophthalmic vein with SOVT (Figures 1, 2). He underwent repeat CT imaging few days later after commencing heparin to monitor his progress and determine ultimate resolution of the superior ophthalmic vein flow obstruction.

Differential diagnosis

This child was initially presumed to have pre-septal cellulitis and was managed (albeit overcautiously) with intravenous antibiotics. One of the reasons for aggressive treatment initially was that the child’s mother reported a significant reduction in his psychomotor activity that was not obvious to the examining clinicians who had not seen him when he was well. The difference was only apparent once the child was better given his background of ADHD.

When the lid swelling worsened despite ongoing antibiotics, and the child developed a painful limitation of up-gaze, imaging was correctly obtained (with contrast) to rule out orbital involvement. It should be noted that at this point there was a very low suspicion of venous sinus thrombosis. In hindsight, one of the peculiarities of the eyelid swelling was that it was soft and “boggy” rather than tense or firm, as might be expected with cellulitis. This may have been an early clue to the aetiology of sluggish venous drainage.

A point of interest here was the absence of conjunctival injection or chemosis. This serves as an important reminder that eye movement limitation is a more important marker of progression to orbital cellulitis or venous thrombosis.

Treatment

The initial phase of presumed pre-septal cellulitis was treated with intravenous co-amoxiclav, which was changed to intravenous ceftriaxone and oral metronidazole. Following the CT finding of SOVT, unfractionated heparin was added on advice of the paediatric neurologists, which was to be omitted just prior to the repeat CT scan (in case the CT revealed an abscess that required drainage). On advice of the paediatric haematologists, this was changed to low molecular weight heparin (LMWH) for long-term management once the CT ruled out a developing abscess.

Outcome and follow-up

The eyelid swelling had remarkably started to improve 24 hours after commencement of anticoagulation, despite no significant improvement on imaging at 48 hours of anti-coagulation. By the third or fourth day of anticoagulation, the swelling had virtually resolved and ocular elevation was nearly full with minimal pain.

Over the next few days and weeks, the paediatric haematologists helped titrate dosage of the LMWH using anti-Xa factor levels. Given that this was a provoked cause of thrombosis, it was recommended that anti-coagulation be given for a period of three months, at which point a magnetic resonance imaging (MRI) or magnetic resonance venography (MRV) would be used to confirm complete resolution of the thrombosis.

Ophthalmology follow-up a month after initial presentation showed complete resolution of eye symptoms, and the child was discharged from the eye clinic. He was followed up as an outpatient by the paediatricians until discontinuation of the anticoagulation.

Apart from some persisting headaches, he was back to his usual energetic self. He joined football training and both he and his mother were pleased with the overall recovery. He was reviewed once more by the ophthalmologists due to persisting headaches; however, no clear cause for these was found.

## Discussion

SOVT is a rare but serious condition that should be considered in certain orbital presentations. It carries a higher morbidity and mortality compared to other more prevalent diagnoses such as pre-septal cellulitis. The aetiology can be non-infectious secondary to inflammation, trauma or thrombophilia for example or infectious due to sinusitis or orbital cellulitis. Infection-related SOVT results in localised irritation and alteration of blood flow within vessels, which can lead to thrombosis in the cavernous sinus or along its tributaries including the superior ophthalmic vein [2,4]. In our young patient, sinusitis was thought to have precipitated the SOVT.

Diagnosis

The impairment of venous drainage that occurs in SOVT results in congestion of the orbital veins, which can present in a number of ways [4]. These include acute painful proptosis, pain on eye movement, swelling of the eyelids and, most importantly, a reduction in the visual acuity [1,4].

Neuroimaging with contrast-enhanced CT or MRA (magnetic resonance angiography) [4,5] is crucial to establishing a diagnosis of SOVT. This can also help to highlight any co-existent pathology that will require intervention such as an infective abscess or cavernous sinus thrombosis [1]. The findings on a CT scan may include engorgement of the superior ophthalmic vein with a possible filling defect and surrounding oedema [6,7]. As per the literature and our experience, once a diagnosis of SOVT is established, it is often successfully managed without serious complications [4,6].

Management

The mainstay of management is medical with anticoagulation, antibiotics and/or corticosteroids [6]. Surgical intervention is usually reserved for when there is an orbital abscess or mass or if the patient has severe Graves' orbitopathy.

Medical management

A multi-disciplinary approach is needed to successfully manage SOVT. Where septic causes are considered, patients should have a full septic screen and be commenced on broad-spectrum antibiotics in line with local microbiology guidelines. In addition to this, anticoagulation is necessary to prevent further thrombosis [3,4].

Surgical management

A surgical approach is considered when medical management alone is insufficient to treat the underlying cause. Examples include drainage of an abscess [1] and optic nerve decompression [2].

## Conclusions

One should be vigilant lest missing this devastating, lethal pathology. SOVT mostly resolves without any sequelae with early detection and management. A focused history and physical examination to rule out orbital involvement and optic neuropathy are critical. Proper management is recommended with broad-spectrum intravenous antibiotics, anticoagulants and surgery, if needed, depending on the aetiology. A key factor behind the successful management of such cases is early intervention and collaboration between the paediatric neurologists, paediatricians and ophthalmologists.

## References

[1] Sotoudeh H, Shafaat O, Aboueldahab N, Vaphiades M, Sotoudeh E, Bernstock J (2019). Superior ophthalmic vein thrombosis: what radiologist and clinician must know?. Eur J Radiol Open.

[2] Sorrentino D, Taubenslag KJ, Bodily LM, Duncan K, Stefko T, Yu JY (2018). Superior ophthalmic vein thrombosis: a rare complication of Graves' orbitopathy. Orbit.

[3] Desa V, Green R (2012). Cavernous sinus thrombosis: current therapy. J Oral Maxillofac Surg.

[4] van der Poel NA, de Witt KD, van den Berg R, de Win MM, Mourits MP (2019). Impact of superior ophthalmic vein thrombosis: a case series and literature review. Orbit.

[5] Lim LH, Scawn RL, Whipple KM, Oh SR, Lucarelli MJ, Korn BS, Kikkawa DO (2014). Spontaneous superior ophthalmic vein thrombosis: a rare entity with potentially devastating consequences. Eye (Lond).

[6] (2023). Superior ophthalmic vein thrombosis. https://eyewiki.aao.org/Superior_Ophthalmic_Vein_Thrombosis.

[7] Walker JC, Sandhu A, Pietris G (2002). Septic superior ophthalmic vein thrombosis. Clin Exp Ophthalmol.

